# "The Hospital" Nursing Mirror

**Published:** 1901-07-20

**Authors:** 


"The Hospital" Nursing Mirror.

Being the Nursing Section of "The Hospital."

[Contributions for this Section of "The Hospital" should be addressed to the Editor, "The Hospital" Nursing Mirror, 28 & 29 Southampton Street Strand, London, W.C.]

### Notes on News from the Nursing World.

### THE MARLBOROUGH HOUSE RECEPTION.

The final arrangements for the reception by the Queen, as President of the Royal National Fund for Nurses, at Marlborough House on Friday, the 19th, are as follows:—The nurses entitled to receive certificates—*i.e.* those whose policies are numbered 7,251 to 8,700, both inclusive—and who have not before been eligible to receive certificates from the President—should be at the garden entrance of Marlborough House at 1.30. The nurses previously eligible, but who have not attended before, or those who have been on active service or plague duty, whether they have attended or not, should be there at 2 o'clock. The gates in the former case will be closed at 1.45, and in the latter at 2.15. As the fullest possible details are sent out with the cards, there is no necessity for the nurses to ask any questions; and by literally following the instructions given they will very considerably assist the marshalling. We may remind the members of the Fund that the offices in Finsbury Pavement will be closed on the day of the ceremony.

### QUEEN ALEXANDRA AND THE JUBILEE NURSE.

At the recent reception by the Queen of Jubilee Nurses one of the members of the Irish contingent found herself short of her badge when the distribution had concluded. She was going away without making any trouble over the incident, but a member of the Royal Family perceived the deficiency, and inquired the cause. Subsequently, the Queen was informed of the matter, and having requested that the nurse should be brought into her presence, conversed with her for some little time, and then presented her with her badge and fastened it on. The *contretemps* should not, of course, have occurred, but, thanks to the graciousness of the Queen, the nurse who had not been provided with a badge went away from the ceremony with a feeling of satisfaction instead of with a sense of disappointment.

### A SISTER AMONG BOER PRISONERS.

On Thursday last week a sister who was in charge of a Boer camp in Ceylon told a number of nurses, who with herself were the guests of the Secretary of the Nurses' Union, that the time spent amongst the Boer prisoners was in many ways a delightful experience. She said that she found them the most pleasant of patients, grateful and enduring, and that the Boer orderlies who assisted in the wards were of the greatest help in nursing, thoughtful and kind in work. Neither was there any bitterness of feeling. The fever was the worst kind of typhoid, and many died, the scenes at the funerals being extremely touching. From a nursing point of view the sister stated that there was a good deal of making shift, and occasionally a hairpin had to serve as a probe, in reference to which the sister told her audience, "You may be sure that I took care it was carbolised well." The doctors, she added, were devoted, and also the nurses who worked under her. English magazines and books were welcomed both by the prisoners in the camp and by the nurses.

### NURSES AND THE CONCENTRATION CAMPS.

It was announced by Lord Raglan in the House of Lords on Monday evening that a committee of ladies are to be sent out to the concentration camps in South Africa to report on the conditions and needs of the refugees. This step is obviously the result of the allegations which have been made respecting the treatment of refugees, but it is a more important matter that there should be a sufficient number of medical men and trained nurses in the camps. Lord Raglan assured the House that both doctors and nurses had been added to the staffs. He did not, however, give any details, and there is an impression that though a few detachments of nurses have lately been despatched to South Africa, the supply is not adequate for all needs. As Lord Raglan admitted, the mortality in some of the camps has been very great. He also conveyed the impression that some of the sickness has been due to ignorance of sanitation on the part of the refugees. But this might have been anticipated and provided for by the employment, for the purpose, of a few of the Army Reserve sisters.

### THE WOMEN'S MEMORIAL TO QUEEN VICTORIA.

On Tuesday afternoon a drawing-room meeting was held in the interests of the Women's Memorial to Queen Victoria, in connection with the Jubilee Nurses, at the residence of Sir John Aird, M.P., Mayor of Paddington, for the purpose of considering the organisation of the borough on behalf of the fund. As Lady Aird, though present, was not well enough to undertake any active work, the chair was taken, at Sir John Aird's request, by Mrs. Melvill Beachcroft, who addressed the meeting on behalf of the fund, the object of which was further explained by Mr. Harold Boulton, and by Lord Mountmorres. On the proposal of Lady Dimsdale, it was resolved to organise the borough forthwith, and a preliminary committee was formed, with power to add to their number. Lady Aird was elected president, and Mrs. Melvill Beachcroft vice-president and hon. treasurer. The latter announced that donations had been received to the amount of £25 from residents in the borough, who had heard of the intention to start a local subscription list. Mr. Shearmer Turner and Mr. Rayner were nominated hon. secretaries for North and South Paddington respectively. A similar meeting was held on Tuesday at the residence of the Mayor of Battersea, Mr. William Davies; and an influential committee was formed.

### PIONEERS OF REFORM IN INDIA.

In the second of the interesting articles contributed by a nurse in one of the Bombay hospitals which appears this week, the writer encourages the hope that she and her colleagues are, in more ways than one, pioneers of reform among the natives of India. She cites some of the difficulties to be overcome in the prosecution of the task, which, though they may afford amusement to outsiders, are enough "to try the patience of a saint" inside. But she is able to speak of the good influence which daily contact with Englishwomen has upon the friends of the patients, and to say that "they are beginning to feel that we really care for them, though they cannot tell why." Her account of her experience of nursing prisoners "who do not feel the least shame for their unfortunate condition," throws light on the nature of the problems which our rulers in the great dependency have to solve; and her account of the manner in which the father of a patient contrived to make in the relapsing fever ward the experiment of "exorcising a demon" by heat furnishes further food for reflection.

### THE AKRILL HOME AT WEST BROMWICH.

The conditions under which the new nurses' home at West Bromwich, whose foundation stone has just been laid, is to be erected, are excellent. Alderman Akrill, of Edgbaston, and formerly mayor of the borough, generously offered to defray the cost of the building, involving an expenditure of £2,000, on the understanding that other people provided an endowment fund of £1,000. The latter amount having practically been subscribed, the home in Grange Road will be available as soon as it can be erected. When it is finished the number of nurses will be increased from three to six, and the staff will be in charge of a matron. From a description of the home which has been sent us we learn that on the ground floor of the building are arranged a dining and sitting rooms for the use of the nurses in residence, matron's room, kitchen, scullery, pantry, linen store, and cloak-room and lavatory, together with the usual out-offices and store-room for bicycles. On the upper floors are eight chambers, two of them being double-bedded rooms, bath-room, two general linen closets, and a large closet for aired linen, warmed by hot water. The building will be entered from the front through a vestibule leading into a capacious hall, from which the staircase ascends. The dining-room is of sufficient size to permit of its being used as a board-room by the committee. The cloak-room will be fitted with the necessary appliances for cleansing infected clothing and for the drying of outer garments. Hot water will be laid on to the bath-room and lavatories, and the scullery is large enough to be used as a laundry. The buildings are designed in the Romanesque style freely treated, faced with dark-red bricks, relieved with buff terra cotta dressings, and the roofs will be covered with tiles. The central portion of the front is carried up as a tower to give the building a distinct public character. The internal woodwork will be of pitch pine stained and varnished. West Bromwich, with its steadily growing population, will thus have no cause to reproach itself for not keeping pace with the nursing requirements of the times.

### FICTIONS ABOUT FEET.

A sweeping allegation is made in a ladies' periodical, which says that "the atmosphere of romance which wraps the nursing profession is cruelly dispelled by the knowledge that most of its votaries are flat-footed." The author of this assertion continues: "More honour to those that they have become so in consequence of their arduous duties; but it is a sad fact, nevertheless, that hospital nurses seldom or never have pretty feet." There are several statements in these quotations which are inaccurate. Save amongst people who know nothing whatever about the subject, there now hangs no atmosphere of romance about the nursing profession. The idea of a nurse's duties consisting of smoothing pillows and brows, and holding cups of sparkling water to fevered lips, in a most becoming costume, no longer obtains credence except with the most ignorant. Secondly, flat-footed nurses are the exception, not the rule. It is a trouble which can generally be cured by ten minutes' daily tiptoe exercise, and it so materially diminishes nurses' powers of endurance that even if at some time during their training they may have suffered from a falling of the instep, few allow it to continue unchecked. Lastly, the contention "that hospital nurses never have pretty feet" could doubtless be easily disposed of by a casual observer at any large hospital. Nurses may not have small feet, but is a foot necessarily ugly because it is encased in a sensible shoe of a size larger than "two's"? We fancy not.

### QUEEN'S NURSES AT NEATH.

The financial position of the Neath Nursing Association was naturally referred to in terms of satisfaction by the chairman at the annual meeting. There was a balance of £45 in hand at the end of the year, and among the contributions were substantial subscriptions from the Neath Corporation, the Neath Board of Guardians, and the Cambrian Lodge of Freemasons. The Post Office staff contributed £1, a collection at the Constitutional Club realised £4 15s., a performance at the theatre £11 15s., the proceeds of a cyclist carnival £34 12s., and an offertory at St. David's Church £15 18s. These figures indicate that all classes of the community are supporting the Association; while the report of the work of the nurses shows that nearly 6,000 visits were paid in the course of twelve months. The necessity of the annual subscriptions being maintained and increased, so as to ensure a regular income, was, however, pointed out. This is always a matter of primary importance.

### A NEW ISSUE AT NEWTON ABBOT.

Nursing questions occupy a good deal of the time of the Newton Abbot Board of Guardians. Last week a new issue arose on the receipt of a letter from the medical officer (Dr. Culross), which was read to the Board, as follows:—"It is with sincere regret that I find it necessary to approach the Board of Guardians with regard to a matter which seriously affects my work as medical officer of the workhouse and the work of the nurses who are under my superintendence. It has come to my knowledge that on several occasions a member of the board belonging to the medical profession, when paying visits to the infirmary, has questioned patients and nurses with regard to the diagnosis and treatment of cases, and has also in some cases commented adversely on my diagnosis and treatment. Your board will understand that such a course of action is not only contrary to medical ethics, but also has a tendency to render more difficult the work of both doctors and nurses. I am writing, therefore, to ask your board to declare definitely by resolution your disapproval of such conduct."

### NURSES AND DIPHTHERIA.

At the annual meeting of the Colchester District Nursing Association Dr. E. A. Hunt, who presided, paid a striking tribute to the capacity of the nurses, of whom he affirmed that they had carried out their work with diligence, skill, and discretion. Dr. Hunt then went on to speak of the services rendered by Nurse Fenning in the hamlet of Old Heath when it was suffering from diphtheria, and said that he wished to publicly thank her for the way in which she assisted him and others. "She proved herself," he added, "clever in deciding what were and what were not necessary cases in which to send for medical assistance;" and he dwelt on the importance of nurses being on the spot whenever diphtheria is prevalent, "in order to go at the immediate call of people to look at suspicious throats."

### BRISTOL GUARDIANS AND NURSES.

The nurses of the Eastville Workhouse Infirmary are at length to have better sleeping accommodation provided for them. It has been finally decided by the Bristol Guardians, with the approval of the Local Government Board, to devote the large ward on the first floor of the hospital building, now used as cubicles for fourteen nurses, to its proper purpose. This will increase the number of beds for patients, and the nurses are to enjoy the advantage of being located in a home. The latter will consist of three houses, which are to be practically knocked into one. Under the new arrangement we trust that a separate bedroom will be placed at the disposal of each nurse.

### PROGRESS AT WARWICK.

We recently alluded to the value of sales of work on behalf of nursing organisations. Another excellent mode of "raising the wind" is by means of rummage sales. At Warwick no less than £66 has been obtained in aid of the local nursing association in this manner. We are glad to see that the collections from the working classes have also been continued in the town, and that during the year they contributed upwards of £8. The number of cases attended by the nurses of the Warwick Association was 132, and the number of visits made 5,492. Lady Warwick, who presided at the annual meeting, and expressed her gratification at the manner in which the movement had been supported, pointed out the need of another nurse. As there is a balance in hand, and the ladies of Warwick are enthusiastic in their support of the association, we do not doubt that the necessary addition to the staff will soon be made.

### THE NURSES OF THE ROYAL FREE HOSPITAL.

Several speakers at the festival dinner in aid of the Royal Free Hospital on Monday evening alluded to the nursing staff. Mr. Burt, treasurer, who mentioned that the most recent addition to the building was the new accommodation for the nurses, said: "I am sure I shall have your sympathy when I say that our nurses deserve all we can give them. No one who visits the hospital will deny that. It is all very well to nurse your friends at home, but when you have a great big dirty drunken man for a patient, or a woman not much better, it is a different thing. Our nurses have to go through all that, and it is done as perfectly as it can be. It is time that our nurses had proper accommodation. We have until recently had cubicles—and anyone who has been at a public school knows what they are—but now we have added some thirty rooms at the top of the hospital, so that each nurse may have a room to herself. Are we not right?" And the audience evidently quite agreed. Dr. Samuel West, senior physician, said he should like to express his thorough appreciation of the nurses' services; it was absolutely necessary to have a good staff, and the question of housing them was one which was bound sooner or later to be faced. He thought it was hardly necessary to say that they had also to be well fed and paid. The Royal Free Hospital had now a most adequate staff, and they were well housed. Another speaker alluded to the matron, who, he said, preferred being at her work among the nurses to dining at the Hôtel Cecil.

### THE START AT CLAY CROSS.

Following the determination of the Clay Cross people to form a nursing association, the work of obtaining the necessary funds was proceeded with, and at a meeting in the Town Hall it was announced that £85 17s. 2d. was available for the purpose. The chairman said that he considered the result of the efforts of the collectors most satisfactory "especially as the times were so very bad." It was then decided to engage a nurse from Queen Victoria's Jubilee Institute, under the direction of a lady superintendent; and the Association very wisely resolved not to receive any complaints against the nurse unless they came through the doctors or the lady superintendent. But we hope that there will not be any.

### PICNICS AND PARSIMONY.

For many years it has been the custom for the nurses at the Ladywell Sanatorium to have a picnic in the summer, but this year a member of the Salford Town Council raised an objection to the custom on the ground that it is not warranted under the Municipality Act, and he received a certain amount of support. The recommendation of the Health Committee, under whose auspices the picnic is held, was, however, adopted by a large majority. We certainly do not believe that the ratepayers will cavil at the trifling expense of an outing which affords a great deal of pleasure to a hard-working staff and can scarcely affect their pockets. It is possible to sacrifice public interests to parsimony as well as to extravagance.

### THE ANGLO-AMERICAN NURSING HOME AT ROME.

Some interesting particulars are given in the report of the managing committee of the Anglo-American Nursing Home, which was submitted to the first general meeting. The purchase of the house in Via Nomentana was effected in June last, and the Home was opened for the reception of patients in November. Up to December 31 seven persons were received as paying patients and two free beds which are provided were occupied for 52 days. As regards the future, the committee—which consists of influential residents—judging by their experience so far, have every reason to believe that the receipts will cover the expenditure. As, however, 38,000 lire, or £1,505, must be paid to complete the purchase within four years; as the expenses of nurses travelling from and to England are heavy; and, if the Home is full, housekeeping expenses naturally increase, the aid of subscribers is invoked to forward the interests of the Home by appeals to their friends visiting Rome and also in England and America. But there is a simple way out of the financial difficulty. It is pointed out that, if every Anglo-Saxon visitor to Rome would contribute only one franc to the fund, the committee need make no other appeal to the generosity of the public. Miss Caroline Kirkpatrick, who has succeeded Miss Gibbons as matron of the Home, is a highly qualified hospital nurse.

### SHORT ITEMS.

At the fifth annual meeting of the National Society for Promoting the Welfare of the Feeble-minded last week, it was stated by Mr. W. H. Dickinson that the expenditure exceeded the receipts to the extent of £600, and that unless the funds increased the excellent work of the Association would be crippled.—On Saturday the ss. *Nubia* arrived at Southampton from South Africa. The following is the official roll of nursing sisters: E. A. Dowse, A.N.S.; B. J. Betty, M. Atkinson, J. E. Phillips, and G. Nutter, all A.N.S.R. All are returning, although Sister G. Nutter is time-expired.

### Lectures to Nurses on Medicine and Medical Nursing.

By NORMAN DALTON, M.D. London, F.R.C.P., Physician to King's College Hospital.

LECTURE XV.—DISEASES OF THE ORGANS OF RESPIRATION—(*Continued from page* 184).

COUGH—EMPHYSEMA—WHOOPING-COUGH—LARYNGISMUS STRIDULUS.

"COUGH" is usually excited by some abnormal material such as phlegm or blood in the larnyx, trachea, bronchial tubes or air vesicles. But it may be excited by nervous influences. For instance, in pleurisy or if the pleura be touched with an aspirating needle, etc., the irritation of the pleura (which covers the lung) causes a cough just as if the irritation were in the lung itself. Also irritation of the pharnyx or the stomach may cause a cough. This is often seen in indigestion. Coughs produced by nervous influences are not attended with expectoration and are therefore called "dry" coughs.

There are certain coughs which are worth describing. There is the "throat" cough which occurs in pharyngitis and is due to the phlegm from the pharynx irritating the back of the larynx. It is associated with tickling at the back of the throat, and is loud, hacking, and frequently repeated. The removal of the phlegm is a difficult matter, and it is effected by the process known as "hawking." The "laryngeal" cough is hoarse or barking. It has been called "croupy." It is associated with hoarseness or even loss of voice (aphonia) and often precedes and accompanies "stridulous breathing." The cough in "emphysema" is attended by a prolonged wheeze. It has often occurred to me that emphysema is an affection which must be very puzzling to nurses. It is so common, and yet there seems to be nothing definite about it. The fact is that in emphysema there is a slow wasting away of the lung tissue. There need be no inflammation. The air vesicles become over-distended with air and their walls simply get thinner until they disappear without leaving any débris. As the chief things in the walls of the air vesicles are blood-vessels and elastic tissue, it follows that, in an emphysematous lung, these two things are deficient. The diminution in the number of blood-vessels obstructs the circulation of the blood through the lungs, so that the blood contains less oxygen; thus the cheeks of emphysematous persons are usually marked by dilated purple veinlets and they become short of breath on the slightest exertion. The loss of the elastic tissue makes the lung less elastic, which causes the act of expiration to be difficult and prolonged, whence the wheezing which is so characteristic of the affection. It will be seen that, although there is wasting of the lung tissue in emphysema, it is a very different thing from consumption, for in the latter disease little tumours, called tubercles, grow in the lung and inflammation occurs. Both the tubercles and the lung tissue then break up and form a lot of débris, which is coughed up mixed with phlegm and pus, while great cavities with rough walls are left in the lungs. Emphysema usually results from repeated attacks of bronchitis and asthma; but it may be brought about by a severe attack of whooping cough. It causes considerable inconvenience to persons who are affected with it; but it may exist in a moderate degree without shortening life.

To return to the different kinds of cough: The cough of pleurisy is so characteristic that once it has been heard it is easily recognised again. It is short and dry, and it excites a sharp pain in the side. Consequently the patient tries not to cough. His efforts not to do so are very noticeable and give the cough a "stifled" or "suppressed" character. The cough produced by irritation in the "stomach" is a loud, reverberating cough without expectoration.

In the disease called whooping-cough, certain remarkable paroxysms of coughing oocur, in some cases only a few times a day, in other cases every hour or even more often. The paroxysm consists in a succession of coughs, which follow each other with such rapidity that the child cannot draw in a breath. Consequently the face becomes congested, the head is held forward, the tongue often protrudes from the mouth, and there is a feeling of suffocation. Finally the child is compelled to draw in a breath, although the larynx is still contracted. It succeeds in doing this by a vigorous effort on the part of the inspiratory muscles, and as the air passes in through the narrowed larynx, a loud whooping noise is made. Whooping-cough is a very infectious disease and lasts from six weeks to two months. The characteristic cough may last for a much longer time than this, but it is thought that infection ceases after the first six weeks. There is always a certain amount of bronchitis present, and in winter, and among the poor, this complication may cause death. Although the paroxysms are very alarming, death almost never occurs during them. It is very important for nurses to know the other complications of whooping cough, so that they may look out for them and report them at once. They are mostly in the nature of accidents, which occur during the violence of the paroxysms. For instance, hæmorrhage may take place from the rupture of blood-vessels. These are most commonly found in the conjunctivæ, but bleeding from the nose and lungs may occur, and, very rarely, a blood vessel in the brain has broken. Occasionally the drum of the ear ruptures and blood may be seen escaping from the outer ear. The air vesicles also may break, and then air gets into the pleural cavity, a condition called pneumothorax. It is interesting to note that except in the case of whooping-cough, pneumothorax is usually fatal, because most often it is a foul abscess or cavity in the lung which breaks into the pleura and sets up a very septic pleurisy. In whooping-cough, on the other hand, the air which enters the pleura has passed through healthy lung and does not excite much pleurisy. Other accidents which may occur during the cough are hernia and prolapsus ani, for both of which a nurse should be on the look out. Lastly, it has been noted that children with whooping-cough often get a sore spot or ulcer at the frænum of the tongue. It is due to the jerking movements of the tongue during the cough and it is a useful sign to the physician, because if he has not heard the cough himself but finds the ulcer in the characteristic place, he feels sure that the case is one of true whooping-cough. The treatment of whooping-cough is directed chiefly towards diminishing the frequency and severity of the paroxysms. Consequently nurses should always count the number of paroxysms and try to estimate their severity by noting their duration and the violence of the coughs. During the paroxysms it may be useful to hold the child's forehead, to fan it or take it to the window in warm weather. Any dyspnœa between the paroxysms would indicate the occurrence of some complication, and should be immediately reported. In rare cases convulsions occur.

It is important for a nurse to distinguish between whooping-cough and a disease called laryngismus stridulous or child crowing. In both a loud whoop or crow occurs, but in laryngismus there is no cough. It is usually associated with rickets; and in many cases certain parts of the bones of the skull, particularly the occipital fossæ, are extremely thin and can be pressed in like parchment. This thinning of the cranial bones is called cranio-tabes. Another curious symptom which may occur in laryngismus stridulus is a spasmodic contraction of the fingers and toes. The thumbs are kept firmly pressed against the palms of the hands, and the fingers are clasped over them. The toes are also sharply bent on to the soles of the feet. Death has been known to occur during a paroxysm of laryngismus; but this is rare, and, as a rule, the tendency to the attacks passes off as the child recovers from rickets.

### A Visit to a Quaker Insane Asylum in Pennsylvania.

By a Touring Nurse.

"Certainly, thee may see our retreat," said one of the managers of the "Friends" Asylum for the Insane at Frankford, a beautiful spot in picturesque Pennsylvania, quite close to Philadelphia, to the writer who was doing a round of American Institutions. He wore his hat while he spoke—according to old Quaker custom—as he did subsequently during a tour of inspection through the wards of this very charming "Friends House" for the mentally afflicted. I believe it is a fact that the Quakers were the pioneers in America as well as in Great Britain, of the humane and kindly *régime* which modern ethics prescribe in the treatment of mental diseases. And my Quaker guide, philosopher, and personal conductor, informed me that his grandfather was among a small body of "Friends" who decided nearly one hundred years ago, when such an idea was a real novelty, to try the effect of peaceable, kindly, and non-violent methods of relieving "Quakers deprived of the use of their reason." The Asylum is situated in about seventy-five acres of lovely grounds, laid out in avenues, with summer-houses, sun-pavilions, "spreading chestnut trees" and green grassland and meadows for the games and outdoor recreations of the patients. All the managers and many of the officials and staff are Quakers, but the patients themselves may be of any creed or sect.

### A Training School for Pupil Nurses.

A training school for men and women pupils is attached to the Asylum, where general nursing is taught in addition to expert and specialised courses in nervous and mental diseases. It is very interesting to know that searching investigation is made as to the personal qualities and temperament of would-be pupil nurses. Some are Quakers, but no hard and fast rule is made that only "Friends" are admitted to the benefits of the Training School. Kindness of heart and imperturbable good temper are, however, required from all nurses who pass the portals of this Asylum of Peace for the unpeaceful and distraught in mind. "Personal kindness to others is the highest human virtue," said my serene companion, who most assuredly practises the gospel he preaches.

### The Watchwords of the Wards.

Gentleness and sympathy are the watchwords of the wards, though a very high professional standard is exacted from the nursing staff in addition to the amiable graces. Private paying patients are admitted, and for these some very pleasant suites of apartments with a private bathroom are provided, or a private patient may occupy with his nurse attendants one of the pretty little cottages in the grounds set apart for this purpose. Art classes are held and technical instruction in wood carving, exquisite embroidery, carpentry, and photography is given to patients whose tastes and skill lie in these directions. I was quite surprised to find the many and varied kinds of amusement provided in this Quaker institution. But, from my observations in the County of Penn, the old school of Quakers, which regarded social pleasures as snares, luring their victims towards the powers of evil, is passing quickly away. And though few among the large community of Friends throughout Pennsylvania would attend a theatre, they constantly join their more worldly neighbours in many kinds of "innocent social relaxation."

### Picnics for Patients.

Very pleasant picnics are arranged for the patients during the summer months, croquet and tennis competitions held, occasional country drives taken, and those who possess artistic tastes are encouraged to join out-of-door sketching expeditions. No theatrical or fancy dress entertainments are allowed, while dancing is still anathema to the Society of Friends. But though my globe-trotting has been somewhat extensive and varied, I have never before visited an asylum where so many charming pursuits and amusements were provided for the inmates. A model gymnasium, fitted with every luxurious appliance for a complete physical training, gives exercise and amusement in wet and stormy weather, and a skilled instructor sees that no patient overtaxes his strength or runs into any kind of athletic danger. All the nurses in turn go through a course of training in the gymnasium during their pupilage, which not only improves their personal health, but gives them a knowledge of the laws of physical culture, which is most valuable to those who later on undertake the care of young mentally-afflicted private patients.

### Tea-parties and Treatment.

The patients and staff are invited to a weekly tea-party, and an "evening sociable," to give the entertainment its rightful American baptism, is arranged once a week, at which popular readings, cinematograph exhibitions and lectures are arranged. In harmony with Quaker tradition, music is somewhat rare. The women patients are encouraged to good works by a weekly sewing class, at which simple homely garments are made for distribution amongst the poor. The entire treatment is designed to make the patients feel that their lives are as occupied, interesting, and useful as though they lived in the outside world. Indeed the Quaker managers profess a wholesome horror of an institutional atmosphere surrounding their good works. In all the homes, hospitals, and asylums belonging to the Society of Friends the depressing spirit of institutionalism is most conspicuously absent.

### Massage and Diet.

They have certainly evolved a most complete and successful training for their asylum nurses; massage is thoroughly taught by a trained teacher in four month courses, and a novelty, so far as insane asylums are concerned, consists in a splendid diet kitchen, at whose head is a competent cookery instructor. Each nurse trained in the asylum must serve a term of three months in this admirable kitchen, where practical instruction is given in diet and all branches of invalid cookery. Examinations are held from time to time to test the progress of pupil nurses, and at the conclusion of the two years' training a final examination decides whether the nurse is qualified to receive the coveted diploma of the asylum school. This certificate ranks high among the many excellent training schools for the nursing of the insane which exist in the United States.

### Hygiene Teaching.

Public and private hygiene teaching of a simple, practical nature is included in the curriculum, and the nurses are impressed with the necessity of striving to be pleasant companions to their patients and taught to be resourceful in inventing new interests for the melancholic and depressed. Patients whose insanity arises from or is associated with alcoholism are not admitted. Since I visited this excellent asylum, which is a model amongst American institutions for the insane, both from its high standard of medical treatment and nursing skill, gardening and other out-door interests and occupations have been added to its curative curriculum. Indeed, the open-air cure for all nervous and many mental diseases is beginning to be an established prescription in American methods of treatment. And in several United States asylums the patients spend the greater part of their time, when the weather allows, either out-of-doors or on open verandahs. All the best American asylums include some kind of winter garden or sun parlour, where even in the worst and most wintry weather, when sitting out-of-doors is impossible, the patients obtain all the benefits of sunshine and light.

### In the Relapsing Fever Wards, Arthur Road Hospital, Bombay.

BY ONE OF THE NURSES.

(*Continued from page* 198.)

### NATIVE NOTIONS OF CLEANLINESS.

Cleanliness is to the ward-boy an unknown quality. He will use the vaseline and wipe his finger on the post that supports the roof. He will place a lamp upon the sister's chair, and when remonstrated with reply blandly, "Káhí chintă náhí! Mí swăchch kăi-ītó" ("You never mind, I will clean it!"); and suiting the action to the words, will rub it violently with a corner of his not over clean "doti" (waist-cloth). He will dry a spoon on the lapel of his jacket and catch up a penholder to stir a powder with, and as we cry out, "Náhí! náhí ăsé năkó kăi-ú' ("No! no! This way don't do"), he replies, with a surprised note of interrogation in his voice, "Năkó" and pops in his finger instead! We remember trying to impress on one who was personally very dirty, the duty of cleanliness. "Dhondoo, you will fall sick if you do not wear clean clothes, you will get fever or plague," but 'twas throwing words away, for with an indulgent smile he answered naively, "I've been working here more than three years, and I've not got sick yet."

### PIONEERS OF REFORM.

When it is remembered that our patients are allowed to have in attendance on them friends of all ages, from little tots of eight or nine to some toothless old man or woman, lame, perhaps, or half blind, all much more dirty in their habits than our ward servants are, some idea may be formed of the enormous difficulty of keeping anything ship-shape. A feeling akin to despair seized us in the earlier days, but we have become more philosophical now and have learned what to expect and, when possible, prevent. And we have the satisfaction of feeling that our wards are in some sort a training school which few leave without having learned something for the common good. Men and women have come in with their sick who seemed hopelessly unresponsive and dull, but in a few days they have learned to help in the bed-making, to feed the patient without shaking him violently by the head in order to induce him to swallow, and to be very helpful in many ways. The masses are being brought into daily contact with Englishwomen. They are beginning to feel that we really do care for them, though they cannot tell why. In this way we are reaching hundreds and thousands of the poor in a manner which they are capable of understanding, and though all we can do for them is but as a drop in the ocean, may we not hope that it is our privilege to be pioneers preparing the way for better things that must surely be in store for the people of this land?

### OUR VISITORS.

Our wards are always open to the public, and we have many visitors. Now it is the Municipal Commissioner with his train of subordinate officials. To the patients and ward attendants he embodies in his person the whole of the mighty "Sercar" or Government, and all preserve a respectful silence, and salaam profoundly as he passes. Next comes the Government visitor, with his bag of rupees, destined to send many a poor waif back to his far-away guam (village) or to keep many a man, in Bombay, from starving until he is strong enough to work once more. The natives call the visitor the piça walà (money man). Indeed, he is a veritable Santa Claus not only at Christmas but all the year round. Biscuits and oranges arrive before him. Toys and pictures follow in his wake. So it is no wonder that old and young alike welcome him with smiles and hearty salaams. Members of the medical profession passing through Bombay on their way from home or up-country are naturally attracted to the hospital, whilst we are occasionally visited by the specialists engaged in making bacteriological investigations, with their cages of monkeys and rabbits, destined for inoculation in the cause of science. Poor little monkeys! One could but feel sorry to connect the thought of sickness and pain with creatures so carelessly gay and so daringly impudent.

### A NEW EXPERIENCE.

Nursing the prisoners was a new experience. Not one of them seemed to feel the least sense of shame for his unfortunate condition. Indeed, most of them by their own account had been sent to jail not for any wrong-doing of their own, but through the fault of others. Almost every day the sentence of one or more would expire; they were then transferred to another ward, where they were free men and relieved from police supervision. Here they often gave us a great deal of trouble by quarrelling with the ward-boys and disobeying rules. A few appeared quite proud of the crime which had landed them in jail. I remember a Brahmin lad who told us glibly that he was in for a C. B. T., which being interpreted meant criminal breach of trust. He had been employed in the Post Office, and had been convicted for tampering with registered letters. This he had done in so clever a way that even his jailer seemed to admire him for it. He was no "common thief" they assured us.

### A TYPICAL PATIENT.

Luxamen, a lad of sixteen, earned his living as an itinerant vendor of papers and magazines. He had a very severe attack of the fever and three or more relapses, so he was with us for a long while after his sentence expired. He had been caught by the police selling stolen property to a jaripurane wâla (native pawnbroker). Of course he had come by it honestly! But he had good points in his character, and was much distressed because his old mother did not know of his whereabouts. He feared that she would be "crying at home." "What books do you sell?" we asked of him one day when he seemed to be in a communicative mood. "*Teet Beets*," he returned, "'*Ome Chat*, *Black an Wite*, and so on. Plenty people buy *Teet Beets*." "How much do you make a day?" "Sometime eight annas, sometime twelve annas, one rupee. Kissmis time I make plenty money. Now," this with a sigh, "this is Kissmis time, and I am here." Poor fellow, it did seem a little hard! Some weeks after his discharge he reappeared at the hospital cleanly and smartly dressed, carrying his packet of books and papers. We did not at first recognise him and told him to go away, but he answered in a tone of pained surprise, "Am not I your patient, Luxamen? I was here so long." It was quite evident he considered that, having been our patient, gave him an indisputable claim upon us, and we had not the heart to disappoint his faith in our good will. He still calls with his pack and we have to be his customers.

### EXORCISING DEMONS BY HEAT.

It will readily be conceived that work amongst a people so simple and child-like, so dramatic in speech and manner has in it a strong element of humour. It is indeed a blessing that it should be so. The work is so big and so sad that without this element it would become almost too depressing. In other hospitals the bulk of the patients recover, but in these infectious hospitals, during epidemic times, very large numbers must die, and in our wards are enacted many pathetic and occasionally revolting scenes. On one occasion the father of one of the patients brought a man to his daughter's bedside to perform certain ceremonies of witchcraft upon her in the hope of effecting a cure. Before their purpose was discovered they had succeeded in smearing her brow with ashes and slightly burning her feet. Burning is a very common practice among these ignorant and credulous people. Delirium is, they believe, due to a demon who may be exorcised by heat. We frequently have patients come to hospital with huge sloughing sores, the result of burns to which they have submitted, in the hope of curing pain or swellings in the head, back or limbs. These neglected sores are very difficult to heal, sadly retard the sufferer's progress, and not infrequently cause death through septic absorption.

(*To be continued.*)

### Fishermen as Nurses.

By a Correspondent.

Fishermen, like bluejackets, make good nurses. On hospital mission ships excellent nursing is often given by deep-sea fishermen to their sick comrades.

Two hospital ships belonging to "the Royal National Mission to Deep-sea Fishermen" are now lying in Yarmouth harbour. I have just had opportunities of inspecting them there and of learning about the nursing in these floating infirmaries.

The new steam-trawling hospital ship, *Alpha*, is beautifully fitted with every possible modern appliance. The swing cots are of the most comfortable kind obtainable. The surgeon's mate, *i.e.*, the sick berth steward, delights in showing to privileged visitors the splendid up-to-date arrangements of this fine trawler.

When a hospital ship is at sea, any sick fisherman afloat—irrespective of creed or nationality—is eligible for admittance. The only qualification is need; and medical attendance and nursing are gratuitous. On a specially constructed stretcher the patient is carried from his rough surroundings to the comfort and quiet of a hospital ship. The cots, which are arranged in rows like beds in a ward, are amidships—the most motionless part of the vessel. Scrupulously clean, and in every way all that could be desired, the relief of the disabled fisherman who finds himself on board the *Alpha* must be great indeed.

There are three other hospital ships belonging to the Mission to Deep-sea Fishermen—the *Queen Victoria*, the *Albert*, and the *Clulow*. But these are sailing ships, and they do not trawl as the *Alpha* does. Each ship carries a fully-qualified medical man, and every mission skipper must hold a certificate for "first aid."

The ship's nurse—the surgeon's mate—is also responsible for the care of the dispensary. The *Alpha* boasts a dispensary that could put many small hospitals to shame, so replete is it with every necessity and even luxury. This ship also carries the X-rays. Often the fisherman-nurse has little to do besides attending to small accidents and injuries but at other times he is very busy.

Not long ago a man who complained of an injured back was taken aboard the hospital ship *Victoria*. Unfortunately, it ultimately proved to be a case of smallpox. There was of course, nothing to be done but for the ship to go into quarantine; and the risk of infection to the rest of the crew was great. In this instance, notwithstanding all the efforts at disinfection and isolation, several of the men caught the disease, though most of them had only slight attacks. But the worst victim was the poor surgeon's mate, who suffered more than anyone. He is badly marked, and bears for life the traces of his devotion to a sick comrade.

Many are the pathetic tales told of the tender unselfishness and skill of these fishermen-nurses. Like other custodians, they have their weaknesses and occasional mistaken ideas of kindness. But who could scoff at the kind thought that prompted a steward to cheer two convalescent patients by playing "Home, Sweet Home" to them on his concertina!

Gentle and kind as women; strong, and often patient as heroes, these toilers of the deep—partly because of the all-round education a seafaring life affords, and partly because of the high-mindedness of which they are so capable—excel as amateur nurses.

### Wants and Workers.

Will anyone having a lady's bicycle for disposal kindly give or lend it to a nurse for a short time to use in a poor scattered country parish? Address, Nurse Hope, High Street, Eynsham, Oxon.

### Crimean Nurses and the Lord Mayor.

One portion of the ceremony at the opening of the Hospital of St. John and St. Elizabeth at St. John's Wood on Monday afternoon was the presentation of distinguished personages in the Board Room. This was very picturesque. The Lord Mayor, clad in his scarlet gown trimmed with black velvet and sable, and holding his three-cornered hat edged with ostrich tips, was accompanied by the Lady Mayoress, the Mayor and Mayoress of St. Marylebone, the Sheriffs of the City, and the City Macebearer and Sword-bearer. The former was in wig and gown, holding the ponderous mace, while the latter, with the sword, wore a brown fur hat with a purple velvet crown. There was also in attendance the City Marshal, who always announces the Lord Mayor in stentorian tones; he wore his gorgeous uniform, with cocktails in his hat. Afterwards, wherever one went, in hospital or grounds, one encountered gloriously clothed footmen in green or crimson velvet gold-embroidered coats, and these bright uniforms formed a striking contrast to the dresses of the two nuns who had been in the Crimean War, and who were presented to the Lord Mayor as "distinguished personages." They wore the black habit of their order, their only decoration being the Royal Red Cross presented by the late Queen. This had the words "Faith, Hope, Charity," and the date. The Lord Mayor said he was very pleased to make the acquaintance of these sisters, and in truth they are women with a most interesting history. Mother Stanislaus and Sister Anastasia went out to the Crimea with the first batch of nurses under Miss Florence Nightingale; they were first at the hospital at Scutari, and then at Balaklava. The privations, Sister Anastasia thinks, were the worst part. They were not trained nurses, and they did not in fact go as nurses at all, but as Sisters of Mercy from the Convent at Bermondsey. They got their training from the wards of the Scutari Hospital, with three other nurses from the same Convent but they must have had an aptitude for nursing, for the choice fell upon these five without hesitation, and one (Sister Anastasia) had only just taken the black veil; she was professed at nineteen years of age. Three out of these five are still living. Mother Stanislaus superintends an orphanage at Walthamstow, and Sister Anastasia works at the Hospital of St. John and St. Elizabeth. One of the guests at the opening of the hospital was the doctor under whom she had her training. It may be added that several of the Sisters of Mercy, on whom the nursing at the hospital devolves, are certificated nurses and were trained at the London Hospital; and that the whole of the nursing staff reside in the villa which forms the front of the hospital in Grove End Road, and is extremely well adapted for the purpose.

### Presentations.

Guy's Hospital.—Miss Hodges, the assistant matron, before leaving the hospital to take up her duties as matron of the Cumberland Infirmary, Carlisle, was presented by the members of the nursing and administrative staff with a pretty silver tea service and oak tea tray; a brass and copper tea-kettle by one of the senior nurses; and a pair of silver salt cellars and spoons by the servants, as an expression of esteem for her work whilst among them, and as a token of their sincere wishes for her happiness and success in her new work.

Down Co. Infirmary, Downpatrick.—Last week Nurse Maud Major was presented at the Down County Infirmary, Downpatrick, with a gold and pearl star-pendant by the Extern students of the Belfast Royal Victoria Hospital on the occasion of her leaving the Extern, where for some time she was in charge, Mr. Moffat and others on behalf of the students personally making the presentation. Prior to her departure from the Royal Hospital Miss Major had been the recipient of numerous gifts, amongst others being a beautiful gold-curb bracelet and padlock, with inscription, from the resident doctors; a silver-mounted crocodile leather dressing-case from sisters and nurses, and photo-frame from theatre sisters. The good wishes of all follow her to her new sphere.

Wisbech Nursing Association.—Miss M. Phillips, "Queen's" nurse at Wisbech, who is leaving that town to take up work at Pontypridd, has been presented with a beautiful travelling clock in a leather case by a few friends in recognition of her services extending over a period of two years

### Echoes from the Outside World.

### AN OPEN LETTER TO A HOSPITAL NURSE.

OF course we are all very much interested in the coming Coronation; but is it not altogether premature to discuss such questions as the route along which the procession will pass and the prices which people will pay for seats to witness it? In the first place, it is officially announced that the Executive Committee appointed to make the necessary arrangements in connection with the event have adjourned until early next year without arriving at any decision, and therefore all statements are purely speculative. Besides, it is nearly twelve months before the ceremony can take place, and if we are going to have "the Coronation route" served up every day in the papers until next June, some of us will begin to get very tired of it. Indeed, I am not sure that it is altogether seemly, at this long interval, to make even imaginary arrangements. As for the attempt to dictate to the King the route of the procession and the period it is to occupy, one really wonders what authority the halfpenny press will next endeavour to exercise. Not content with trying to conduct the war in South Africa, control the Mediterranean Fleet, frame the Budget, and dictate the foreign policy of the nation, some of these little sheets edited by little people in Fleet Street offices will presently propose to grasp the reins of sovereignty, and hang everyone who does not obey their mandates.

---

IT may interest you to hear of the gift which the ladies of Natal are going to present as a souvenir to the Duchess of Cornwall and York when she arrives at Durban. Ten of the principal ladies in the colony have undertaken to get together £10 each by house-to-house collection, and as the strain on everyone's purse in the colony has been pretty considerable the last year or two, to obtain the necessary money involves many long walks and tiring, up-hill climbs. The £100 is to be spent on a gong. The base is to be of Natal wood on which are mounted two hippo-horns which are bent over from the sides so as nearly to meet in the centre. From these hang three cases which originally contained pompon shells. One is inscribed Ladysmith, another Colenso, and a third Spion Kop, each having, of course, been picked up in the locality with which it is labelled. The gong striker is of gold from Zululand, the quartz all being Colonial. I should fancy that the Duchess will be pleased with the offering, because it has the distinct merit of being well thought out, original, and up-to-date.

---

THE report of the Select Committee of the House of Lords on the early closing of shops, which was issued on Tuesday, is a document of considerable importance and significance. Whatever may be urged against the principle of compulsion, it is inconceivable that the statement of Lord Avebury and his colleagues, that the present hours of shopkeepers and shop-assistants "are grievously injurious to health, especially in the case of women," will be ignored either by Parliament or by the public. You may remember that, when Lord Avebury asked for the appointment of the committee, Lord Salisbury, in agreeing to the proposal, more than hinted that its deliberations would not be followed by legislation. But as he himself first consented to join the committee and then signed the unanimous report, it must be assumed that the evidence given has modified, if not entirely altered, his views.

---

FOR the present, at all events, there are not to be any female solicitors in Scotland. Miss Margaret Strang Hall, the plucky girl of eighteen who has for some time knocked at the doors of the profession, has been unsuccessful in her appeal. You will remember that she sought entrance to the Law Agents' Examination, with the idea of being articled to a solicitor at Troon and was refused. With commendable persistence she then petitioned the Court of Session at Edinburgh, and Lords Adam, Kinnear, and Pearson have now signified that they are in accord with the other judges who had been consulted, and have dismissed her petition. They declared, in fact, that the law must be altered before a woman can be allowed to practise as a solicitor. It is not easy to get the law altered, but Miss Hall has youth as well as time on her side, and she need not by any means abandon hope. There are members of the House of Commons who would certainly vote in favour of the admission of her sex to a profession which some few ladies, though not, I think, many, might adorn.

---

WHEN tennis, and nothing but tennis, was the rage alike in town and country there were found many enthusiasts whose faces showed a superior sneering smile when the word croquet was mentioned, and who audibly pitied the poor old-fashioned folks who continued to play so antiquated a game. But, though croquet took a back seat, it never succumbed to the contempt poured upon it, and now it has risen up again a greater favourite than ever. It is only natural that it should do so, because to play it well requires not only practice and dexterity, but also the power of looking ahead and calculating the effect which one stroke will have upon another. Moreover, it is a game which can be enjoyed equally by the old as well as the young, though now that the regulation mallet is so much heavier than that used in the old-fashioned game, it requires rather a strong arm to drive the ball into the desired position.

---

THE new rules have, to my mind, a great many advantages over the old. The most important is that it is impossible to be called upon at a croquet party to spend a couple of hours wandering disconsolately about the lawn waiting till seven other players have finished their turns, for, as in tennis, four is now the ideal number, though very often two players take two balls each, and play the match in that way. Again, the exasperating practice of sending an enemy time after time into a far distant gooseberry bush, or behind a tree, from which the poor victim had to return over a half-mown lawn, or across gravel paths, feeling far from amiable, is no longer allowed. A player can still put an antagonist quite out of a line for her hoop, but she must not send her outside the white chalk line which now surrounds a croquet, as well as a tennis, lawn. Should she by accident do so, it is she herself who loses most by the evil stroke. At fashionable little croquet gatherings there is no chance of a couple of players occupying the lawn all the afternoon—a game of two competitors, with two balls each, has been known before now to occupy five hours—as the hostess arranges all beforehand. A couple are allowed to play for fifteen or twenty minutes as may be determined, and at the end of that time, the player who has gone though most hoops is declared the winner, and she may play again, but the unsuccessful lady retires from the field. Thus, a tournament of, say, eight or ten players can be easily concluded in a single afternoon. Of course it is correct as in all other games played by grown-up people nowadays to award prizes. No one is expected to compete solely for the love of the game.

---

LOOKING down the streets on a hot day, it is difficult to realise that three years ago any one driving a horse with a hat upon its head would have caused considerable surprise, even if he had not been hooted or jeered at. This summer it is quite an ordinary thing to note at least every third horse with his head protected; and an elegant victoria with a lady in muslin and transparent lace, drawn by a couple of prancing horses in decorative hats, the coachman and footman wearing Panamas, is by no means an unusual sight in Piccadilly or Bond Street. The fashionable saddlers keep horses' hats in three or four different shapes—sometimes adorned with rosettes or bows—so that even a horse can have the one best suited to his particular style of beauty. Luton has found that the demand is so considerable for horses' hats that it is necessary for the leading manufacturers to include them in their latest novelties, and next year is likely to find these articles everywhere in great variety. Many private persons who are especially fond of animals make a point of giving equine hats to such owners of horses as are, or think they are, too poor to buy for themselves, and a humane society for the purpose of providing costermongers and itinerant sellers free of cost has also been started.

### Everybody's Opinion.

(Correspondence on all subjects is invited, but we cannot in any way be responsible for the opinions expressed by our correspondents. No communication can be entertained if the name and address of the correspondent is not given, as a guarantee of good faith but not necessarily for publication, or unless one side of the paper only is written on.)

### RE A SHABBY CORPORATION.

"THE MATRON" of the County Borough Fever Hospital, Halifax, writes: Will you allow me to say that (Mrs.) Sarah Ann Moore was not engaged as a nurse, but as a washerwoman at 4s. a day. She was engaged by her own son, who is caretaker of the Luddenden Joint Hospital, to where the smallpox case was removed. A trained nurse and probationer nursed the case. The Halifax Corporation are in no sense of the word shabby to those taken ill whilst performing their duties, be they either nurses or servants. I do not wish to enter into the details of the case, but I do think it my duty to correct the idea given by you that nurses here are engaged as servants and are not well treated.

[We gladly insert the matron's letter. Our remarks, however, were entirely founded on a report in one of the local papers of the proceedings at the County Court, according to which the Judge ordered the Halifax Corporation to pay the claim in full.—ED. HOSPITAL.]

### PRIVATE NURSES' FEES.

"A. B." writes: Nurses nowadays are looked on as a necessary benefit by the public, and yet we frequently hear of how "dreadfully expensive a trained nurse is." Why should this be so? "Homes," to which both doctors and the public invariably fly when a nurse is required, are generally made up of "salaried nurses" receiving on an average £30 a year. Such nurses are sent to all kinds of cases and the highest possible fee charged for them by "the home." Whenever the nurses grumble (nurses have a knack of grumbling occasionally) and say how hard they work for their salary the answer is, "Oh, the slack time is coming." That time, however, they find generally belongs to the nurses who "take their own fees," if a salaried nurse is available. Now surely both the public and the doctors could alter all this! Is there no redress for a patient who must have a trained nurse, but who cannot afford the two guineas charged by "the home"—two guineas, of which the nurse only gets 12s. 6d.? It is not nurses who are so expensive, but the homes that are to be blamed for the common idea that trained nurses get exorbitant fees. I suggest that both trained nurses and doctors join hands and form homes on some more reasonable lines as regards both the amount the patient pays and the amount the nurse receives for her services, to be known, say, as "Queen Victoria's Registered Nurses' Homes."

### DISTRICT NURSES AND MATERNITY NURSING.

"T. C. E." writes: I should much like to hear your opinion on the question of the advisability of allowing a district nurse to take maternity nursing and the dressing of wounds, as there seems to be so much diversity of practice with regard to it. The Queen's Nurse who gave an interesting little account, a few weeks back, of a day's work, speaks of dressing wounds in the morning, and making an examination per vaginam in the evening, as though it were quite a common occurrence. On the other hand, I know a district nurse who takes maternity cases, and who will never do more than direct the dressing of bad legs, etc. She says she is sometimes charged with being too grand to attend to these chronic cases, and one can hardly wonder, if, as is likely, the people know that other nurses in a like position do it.

[In this as in so many other matters much depends upon the possibility of maintaining asepsis. Nurses must not imagine that because they see surgeons pass from case to case in hospital operating theatres that they can do so with impunity in the dwellings of the poor. It will be found in practice that it is a bad plan for a district nurse to attempt to combine midwifery with her general work. First of all it is a dangerous practice; then the knowledge that she is expecting a midwifery case must constantly hamper her in regard to her proper district work; while the fact that she takes midwifery cases must stand very much in the way of properly trained midwives settling and working in any district where such a nurse is established.—ED. HOSPITAL.]

### The Nurses' Bookshelf.

OBSTETRIC AND GYNECOLOGIC NURSING. By EDWARD P. DAVIS, A.M., M.D. (London: W. B. Saunders & Co 1901. Price 8s. net.)

THIS is a book of 400 pages, and at first sight it may appear that so large a volume can hardly be necessary in dealing with but one portion of a nurse's work. But a careful investigation of its contents shows that there is but little padding, and that the size of the volume is due to the fulness of the directions given, and perhaps, here and there, to some repetition, which is inevitable if each section is to be complete in itself. The first half of the book is devoted to obstetric nursing. A good account is given of the growth of the ovum, of the changes which take place in pregnancy, and of the stages and events of labour. But it must be understood at once that it is not a handbook of midwifery, and no directions are given as to the conduct of labour, except so far as is necessary in order that the nurse may know what to do in case the child should arrive before the doctor. Beyond this the pages are entirely occupied by purely nursing subjects, it, of course, being understood that the author does not content himself with merely saying what is to be done, but takes considerable trouble to explain the reason why. In many points the proceedings described differ somewhat from the methods generally adopted in England; as, for example, in regard to the frequent use of the dorsal position even in the application of forceps. From beginning to end the author insists upon the most careful use of antiseptic measures, and perhaps one may say that the keynote of the whole is struck when he says that "the nurse should consider each pregnant and parturient patient as a surgical patient, and so far as antiseptic precautions are concerned an abortion or labour must be treated as a surgical operation" requiring the strict observance of those antiseptic methods which are invariably practised by careful surgeons. "She should not insert her finger or an instrument within the genital tract of the patient without the consent of the obstetrician, for the same reasons which would prevent her from inserting her finger into a wound or into the abdominal cavity during an operation." This is undoubtedly the proper line to take, and this is taken consistently throughout, both in the obstetrical and the gynecological sections. In fact, there is no broad line of distinction to be drawn between the nursing in gynecology and in operative midwifery. The descriptions of the work expected of the nurse in regard to the various operations are very clear, but full as are the details given, the limitations of a nurse's duties are strictly insisted upon; the tone of the book is good; and we have no doubt it will be found very useful.

THE DISTRICT MIDWIFERY CASEBOOK. By Sister MARGARET CALEY, Matron of the Fulham Midwifery Training School. (London: Shepherd's Printing Works, Walham Green. Price 4½d.)

MATERNITY nurses will welcome Sister Caley's little casebook, sold at the modest price of 4½d., which has pages divided clearly for ten days' attendance for each case, and with a temperature chart. Even blotting paper for each sheet has not been forgotten. What would Sairey Gamp say to such "fine work." It will be remembered that she declared that washing daily of the face and hands could not be legitimately demanded for the payment of 2s. 6d. per day. It is very helpful to have a record of what nurses were present at the confinement, and who finally attended the case, should there be any need for inquiries afterwards, and this reminder is so admirably arranged that it all but records for itself. The only thing that occurs to us as an improvement is that the book should be interleaved with plain pages to note any peculiarities in the case.

THE COOK'S DECAMERON. A Study in Taste, containing over two hundred recipes for Italian dishes. By Mrs. W. G. Waters. (London: William Heinemann. 1901.)

THERE are many ways of serving food, as may be gathered from any ordinary cookery book. Nowadays even the recipes must be served up in dainty fashion, garnished, maybe, with philosophical reflections or strung together on a little story. In this case it is a very little story. All the same we fully agree with some of the ideas it is intended to inculcate, especially as to the possibility of producing tasty dishes from simple materials by aid of careful cooking and the judicious use of herbs and vegetables for giving variety of flavour. As for the recipes, they are no doubt very good and in a sketchy and suggestive way will be found of use by some. Whether anyone except a good cook would make anything of them is another matter. Take for example the following for a "Pollastro in Fricassea al Burro." "Cut up a fowl, and cook it in a fricassée of butter, bacon, ham, herbs, mushrooms, truffles, spice, and good gravy or stock. Serve in its own gravy." Nothing could be nicer, but it well illustrates the broad distinction which must be drawn between a handbook of cookery which tells how to do things, and a book of recipes which only tells what things to do. Hence the full appropriateness of the name of this little book. It is the Cook's Decameron. One must first of all be a cook. As for the Decameron side of the business one can only say, Poor old Boccaccio! Still, it is a brightly enough written little book—the oddity is that such-like rhapsodies on tastes and flavours should find an audience, which they clearly do.

### Appointments.

CLAPHAM MATERNITY HOSPITAL.—Miss Margaret Pattison has been appointed matron. She was trained at Bolton Infirmary, and subsequently held the posts there of theatre sister and night sister. She was then charge nurse at the Royal Halifax Infirmary, and sister at the Royal Hospital, Portsmouth, where she took the assistant matron's holiday duties, and for the last year has been matron to Rudgwick Sanatorium, Sussex. She holds the L.O.S. certificate.

ROYAL ALBERT EDWARD INFIRMARY AND DISPENSARY, WIGAN.—Miss A. M. Oslar has been appointed assistant-matron. She was trained at the Royal Albert Edward Infirmary, Wigan, and since the completion of her training has held the appointment of sister to a block of male wards, as well as that of night superintendent.

SEAHAM HARBOUR INFIRMARY.—Miss E. A. McLaren has been appointed matron. She was trained at Rotherham Hospital, Yorks, and has since been charge nurse in Lewisham Fever Hospital, London, and nurse-in-charge of the sick wards of Lanark Combination Poorhouse, N.B.

### To Nurses.

WE invite contributions from any of our readers, and shall be glad to pay for "Notes on News from the Nursing World," or for articles describing nursing experiences, or dealing with any nursing question from an original point of view. The minimum payment for contributions is 5s., but we welcome interesting contributions of a column, or a page, in length. It may be added that notices of entertainments, presentations, and deaths are not paid for, but, of course, we are always glad to receive them. All rejected manuscripts are returned in due course, and all payments for manuscripts used are made as early as possible after the beginning of each quarter.

### For Reading to the Sick.

THROUGH DEATH TO LIFE.

FROM vintages of sorrow
Are deepest joys distilled:
The cup outstretched for healing
Is oft at Marah filled:

God leads to joy through weeping,
To quietness through strife,
Through yielding unto conquest,
Through death to endless life:

Be still; He hath enrolled thee
For the Kingdom and the crown;
Be silent; let Him mould thee
Who calleth thee His own.

Such silence is communion,
Such stillness is a shrine,
The "fellowship of suff'ring"
An ordinance divine;

And secrets of "abiding,"
Most fully are declared
To those who with the Master
Gethsemane have shared.

*E. S. Elliott.*

Death has two sides to it—
One sunny, and one dark—as this round earth
Is every day half sunny and half dark.
We on the dark side call the mystery *death*;
They on the other, looking down in light,
Wait the glad *birth*, with other tears than ours.

*George MacDonald. A Hidden Life.*

Death—what is it to him who is ready? Is it not a visit of an angel come to bring him "good tidings of great joy" —come to call him from pain and weakness and sin and sorrow to perfect rest and cloudless peace? To the servant who is watching, Death is but the messenger of his dear and loving Lord come to take him home. It is but the narrow stream which parts him from the promised land—the little golden gate which opens into Paradise.

*Bishop Walsham How.*

You who aspire to Eternity will quickly comfort yourself for the adversities of this life, which last for but a few short and passing moments. In this Eternity we shall enjoy unbrokenly the society of our loved ones without fear of separation for ever. Dwell in this blessed and glorious thought. Since our Saviour has died, every death is happy to those who die in the faith of the Lord Jesus. Whoever thus trust in His merits will not give themselves up to overwhelming grief for the death of those whom He has bought and received as His own.—*S. François de Sales.*

These hours, and that which hovers o'er my end,
Into Thy Hands, and Heart, Lord, I commend.

Take both to Thine account, that I and mine
In that hour and in these may be all Thine.

That as I dedicate my devoutest breath
To make a kind of life for my Lord's death;

So from His living, and life-giving death,
My dying life may draw a new and never-fleeting breath.

*R. Crashaw.* 1646.

### Notes and Queries.

**The Editor is always willing to answer in this column, without any fee, all reasonable questions, as soon as possible.**

**But the following rules must be carefully observed :—**

**1. Every communication must be accompanied by the name and address of the writer.**

**2. The question must always bear upon nursing, directly or indirectly.**

**If an answer is required by letter a fee of half-a-crown must be enclosed with the note containing the inquiry.**

*The Finsen Light Treatment.*

(144) The Dowsing Radiant Heat Company, 24 Budge Row, Cannon Street, write:—We notice in your issue of July 13th, under Notes and Queries, a question as to where the Finsen apparatus and treatment could be obtained in London, and the answer that it is only to be obtained at the London Hospital. We beg to state that we have the Finsen apparatus at our West End establishment, 28 York Place, Baker Street, and we have already advertised this in your special number. At any rate, we have pleasure in enclosing particulars of same; we should be much obliged if you would kindly note same.

*A Maternity Engagement.*

(145) Will you kindly tell me what I can do under the following circumstances? I am a trained maternity nurse. I was engaged to nurse a lady from June 1st. It is now July 14th, and still no signs of being wanted. I have had to refuse three offers of work in consequence. I have a case to go to on August 12th, if not wanted before. If the previous case does not come on before, can I claim any compensation? I was going at a much reduced fee, and during all that time I have had my lodgings to pay for and other expenses to meet, which I should not have done if at work.—*Nurse Emmeline.*

We believe that you are entitled to a fee and board wages for the time for which you were engaged, dating from June 1st. Probably your best plan would be to send in your account. Of course your engagement for August stands.

*Domestic Medicine.*

(146) Can you recommend me a good book on domestic medicine, one giving the symptoms, causes, and treatment of disease?—*M. J.*

"A Manual of Family Medicine for India," by Sir William Moore (J. and A. Churchill); this, although written for India, is a good general handbook. So far as children are concerned, Chevasse's "Advice to a Mother" is a useful book (J. and A. Churchill).

*Sterilising.*

(147) Will you kindly tell me if there is any way of sterilising instruments without turning them black? What can I put into the water?—*Sister Mary.*

One per cent. of sodium carbonate should be added to the water in which the instruments are to be boiled; that is nearly 5 grains to the ounce.

*Washing Babies' Eyes.*

(148) Would you kindly inform me how boracic lotion should be made for washing babies' eyes, and what strength?—*A. M. S.*

Ten grains or more to the ounce. A saturated solution of boric acid in cold water contains just over 19 grains to the ounce, so that if a little hot water is added to a saturated cold solution we have what is wanted. It may be questioned, however, whether simple boiled water allowed to cool is not just as good.

*Phthisis.*

(149) Will you kindly tell me if there is any institution in the north of England where a young man suffering from phthisis can receive the open-air treatment? He can only afford to pay a moderate sum per week.—*B. W.*

A list of institutions where the open-air treatment is in operation was published in THE HOSPITAL of April 20.

*Training.*

(150) My daughter has entered the New Victoria Hospital, Liscard, for a three years' course of training. I have been told that the value of a nursing certificate depends upon whether or not the school maintains a resident medical officer, and I should be glad to know if this statement is correct, as the above school does not fulfil this training condition.—*J. W. S.*

The Local Government Board requires that all applicants for the post of superintendent nurse of a workhouse should possess a three-years' certificate from a school maintaining a resident medical officer. Therefore the certificates of the New Victoria Hospital will not be a sufficient qualification for this branch of nursing, nor will it carry any weight for important appointments elsewhere.

*Homes.*

(151) Can you inform me of a home for an old lady of seventy, who is blind and paralysed?—*Interested.*

Can you tell me of a home for an elderly lady, paralysed and almost helpless. She can pay a little?—*F. M.*

Can you tell me of a home where an infirm childish old lady of eighty-five could be received? She could pay 15s. weekly.—*Nurse E.*

The British Home and Hospital for Incurables, Crown Lane, Streatham. Office 72 Cheapside, E.C. The idiotic, the insane, and the blind are ineligible. Home for Confirmed Invalids, 36 Aubert Park, Highbury. Royal Hospital for Incurables, West Hill, Putney Heath, S.W.; office, 106 Queen Victoria Street, E.C. St. Peter's Home and Sisterhood, Mortimer Road, Kilburn, N.W. The Woodside Home, Whetstone, N.

*Medico-Psychological Society.*

(152) Will you kindly tell me where the lectures for the Medico-Psychological Society are held, if they are free; and what books are required to be studied for the examination?—*Nurse M. L.*

Apply for full particulars to the Registrar, 11 Chandos Street, W. The next examination is on the first Monday in November.

*Employment.*

(153) 1. Can you tell me if probationers who have had a year's training at a workhouse infirmary are accepted as probationers at the London Hospital? 2. What hospitals offer appointments on the nursing staff to nurses who have had three years' infirmary training?—*Constant Reader.*

1. Write direct to the Matron of the London Hospital, and give full particulars. 2. It is impossible to say.

*Time for Disinfection.*

(154) I have been unfortunately called in to a case of puerperal fever. After seven days my patient was sent to a hospital, and I should be exceedingly glad if you will tell me how long a period ought to pass before it would be right for me to attend another case? Of course after I had thoroughly disinfected my clothing, &c.—*Nurse N.*

A month is the time usually mentioned, but much depends on the extent of the disinfection of hair and clothing as well as of person.

*Baden Powell's Nurses.*

(155) Could anyone wishing to become one of Baden Powell's nurses enter his staff as probationer, or must one be a trained nurse before joining?—*Maud B.*

How can I obtain full particulars of Baden Powell's nursing staff? 2. Can you tell me of any other agency by which nurses may obtain work in South Africa other than the Army Nursing Service Reserve?—*Miss B.*

*Maud B.* You must be trained first. *Miss B.* Write to the authorities in South Africa. You may obtain particulars of nursing prospects in South Africa from the Emigrants' Information Office, 31 the Broadway, Westminster, and if fully qualified might obtain work through the Colonial Nursing Association, Imperial Institute, S.W.

*Uniform.*

(156) Will you kindly tell me if I may wear my nurse's uniform? I have been in hospital nine months, and was obliged to leave on account of illness. I am now nursing an old lady. I have still my uniform as it was my own.—*S. W.*

You should not wear the uniform of a hospital after you have left it.

*Club.*

(157) Can you tell me if there is a woman's club anywhere within distance of this infirmary?—*M. J.* (*Shoreditch*).

The East End Club (ladies), 57 Whitechapel Road, E. Hon. Secretary, Miss G. Moseley, might suit you. For list of ladies' clubs see the "Englishwoman's Year Book."

*Turkish Bath.*

(158) Do you think it advisable and desirable for anyone not under medical advice to buy a Century Thermal Bath Cabinet, with the object of taking Turkish baths at home?—*Epicene.*

If in good health this depends upon the state of your pocket, if in bad health you should consult a doctor.

*Dispensing.*

(159) I intend to become a nurse, not a dispenser, but having a little time to spare before beginning my probation, I should like to know if a knowledge of dispensing will be an advantage to me later.—*Aspirant.*

I am a matron of some years' standing, and now think of taking up dispensing. Will you tell me if there is a good demand for assistant dispensers; and what is the usual salary for one holding the assistants' certificate of the Apothecaries' Hall?—*M. E. M.*

Will you kindly tell me what examinations are necessary to become a dispenser? Can I prepare at home, and what books would be useful?—*E. A. L.*

*Aspirant.*—Knowledge is always useful; and time and opportunity permitting, you would do well to study dispensing. *M. E. M.*—The salaries of assistant dispensers range from £40 to £60 a year; and there is a fair demand for the services of such amongst medical men. But the certificate of the Pharmaceutical Society, 17 Bloomsbury Square, is a necessary qualification for any important position. *E. A. L.*—Write to the Society of the Pharmaceutical Society, 17 Bloomsbury Square, W.C., for all particulars. You cannot be prepared for the examinations at home.

*Weak Intellect.*

(160) Will you kindly tell me of a home where, for a small fee, we could place my brother of 17 who is weak in intellect?—*Nurse Marion.*

Write to the Secretary, the National Association for Promoting the Welfare of the Feebleminded, 53 Victoria Street, S.W.

*Advice.*

(161) I have been nursing twelve years, and I would like to take a small house in a country village in Essex as a home for nurses who could pay 10s. a week for board and lodging. There would be a large garden, every home comfort, and no rules. Please tell me if you think this would answer.—*M. G.*

It would certainly not answer. Every home must be conducted by rule; and a charge of 10s. a week is not enough to provide for the fixed charges of the house as well as the current expenses of housekeeping. You must have, in addition, either private means or capital behind you.

*Red Cross.*

(162) Will you kindly tell me how I may become a member of the Red Cross Society?—*M. R. G.*

Through the Army Nursing Service Reserve. Apply the Hon. Secretary 18 Victoria Street, S.W.

*Qualification.*

(163) I have had three years' training in an asylum, and possess the certificate of the Medico-Psychological Society. I have also had one year's training at St. Mary's. Do you think I shall have any difficulty in obtaining a situation as matron of an asylum?—*M. A. B.*

You are sufficiently qualified to take charge of a small asylum. Many institutions now, however, insist on a three years' training in general nursing as well as the experience in mental work.

**Standard Books of Reference.**

**"The Nursing Profession: How and Where to Train." 2s. net; post free 2s. 4d.**
**"Burdett's Official Nursing Directory." 3s. net.; post free, 3s. 4d.**
**"Burdett's Hospitals and Charities." 5s.**
**"The Nurses' Dictionary of Medical Terms." 2s.**
**"Burdett's Series of Nursing Text-Books." 1s. each.**
**"A Handbook for Nurses." (Illustrated.) 5s.**
**"Nursing: Its Theory and Practice." New Edition. 3s. 6d.**
**"Helps in Sickness and to Health." Fifteenth Thousand. 5s.**
**"The Physiological Feeding of Infants." 1s.**
**"The Physiological Nursery Chart." 1s.; post free, 1s. 3d.**
**"Hospital Expenditure: The Commissariat." 2s. 6d.**

**All these are published by THE SCIENTIFIC PRESS, Ltd., and may be obtained through any bookseller or direct from the publishers, 28 and 29 Southampton Street, London, W.C.**

### Travel Notes.

BY OUR TRAVELLING CORRESPONDENT.

### LXXVI.—FROM ALF TO TREVES.

BETWEEN Cochem and Eller the river makes a large loop covering some 12 or 14 miles; it is a particularly beautiful spot and may be visited either on foot or by the steamer; the rail cuts off this great bend by means of a long tunnel. Alf is our destination, which is reached by a ferry from Bullay; it is a primitive boat, and sometimes is inclined to stick fast on the shallows. There is so much to be seen round Alf that one hardly knows where to begin; the favourite excursion of a mild kind, occupying an afternoon, is to the Marienberg.

### THE MARIENBERG AND PÜNDERICH.

Alf and Pünderich lie close together at the two ends of a loop of the river which extends over seven miles; the pleasantest way is to walk up to Marienberg (it only occupies half an hour), and there have coffee in the inevitable restaurant. These perpetual reminders of creature comforts in Germany are certainly convenient, but far from romantic. In every possible and almost impossible spot the wily Teuton places a restaurant and the amount of money so gained must be prodigious. The ruins are partly those of a castle and partly of a large nunnery erected here in the twelfth century; very little remains however. From the Marienberg walk down in ten minutes through the vineyards to Pünderich, where you can take the steamer if hours fit, or walk the seven miles back to Alf. The road is very good, and, if not hurried, the walk is delightful.

Another pleasant way home but rather longer is to descend to Bertrich and thus home; this would make, perhaps, a round of eight miles from Marienberg and the road is difficult to find, but worth taking a little trouble to puzzle out.

### ROUND THE ELLERER BERG.

There is a great deal to be seen in this loop of the river between Cochem and Eller. One of my favourite villages is Bruttig, which has a steamboat pier. It is rich in quaint corners and mediæval houses. Then there is Beilstein with the ruins of a huge castle above; Senheim, and, above all, Ediger, where there are old walls and fortifications—a mine of wealth for artistic purposes.

Bullay, itself, immediately opposite to Alf, has some delightful walks and the celebrated view of the "four lakes" seen from the "Vierseenplatz." From here, too, there is a grand view of the Marienberg.

Zell should be seen in an afternoon's excursion. It has an interesting castle. From Kaint, opposite to Zell, there is a path up to the Marienberg.

### TRABEN AND TRARBACH.

You will notice that these two places are on the railway—at least Traben is; but then there is a reach of river all the way to Berncastel unattainable by train, so that to visit it you must use the steamer or walk. It is, of course, better to visit Traben and Trarbach from Alf and explore the loop from Berncastel. Behind Trarbach lies the beautiful wooded valley of the Kautenbach-Thal. A very nice excursion for good walkers is from Trarbach across to Berncastel, passing Wildstein and Kautenbach. This is a long walk and occupies more than three hours, but a much more direct road between the two places is not more than three or three and a quarter miles. Round the river loop there are many picturesque villages—Cröff, with a very handsome timbered house—Uerzig and Rachtig, too, are worth visiting.

### BERNCASTEL AND THE TIEFENBACH-THAL.

Berncastel is very beautifully situated, and, like all the Moselle towns, is picturesque and old-world. I rather advise you to settle here, because there is a local steamer to and from Trèves, which is an immense advantage, so that if you are unable to stay in that wonderful old city you can pay it a visit from Berncastel, taking the steamer in the morning and returning by train in the evening, so as to have more time at your disposal.

There are lovely walks to be taken in the Tiefenbach-Thal of varying lengths, among others to Mouzenfeld and Veldenz. I do not think the river is quite so interesting above Berncastel as below, but there is much to be seen all the same; the small villages between Berncastel and Trèves have all some point of interest either in themselves or in the surrounding country. Mühlheim lies at the mouth of the Veldenz valley mentioned above and affords charming walks, while Winterich is close to the slopes of the Geiersberg, which form a striking picture. Pisport is celebrated for its wine, and has been for many centuries. One mile above Pisport lies Ferres with the rapid little Throm flowing through a village of that name.

### ON THE MAIN LINE AGAIN.

It is worth while to reach Wengerohr and so take up the main line to Trèves. From Wengerohr you can visit Wittlich, a little to the north, or proceeding further up go to Salmohr, where there is a fine abbey $1\frac{1}{2}$ miles off at Eberards-Clansen. Observe the carved altar here. Very beautiful walks may be found round about, and helping yourself by the train, long distances can be covered. Another day I must say a little to you of Trèves, one of the most interesting towns in Germany.

### THE VOLCANIC EIFEL.

This really is suitable as a holiday ground by itself, but if this is not practicable and you are anxious to have a glimpse of that curious part of the world, start from Alf via Bertrich, passing Houtheim, Strotsbüsch and Römersberg to the Pulvermaar which is one of the large crater lakes. This can all be done by carriage and makes a drive of $8\frac{1}{2}$ miles each way. If you propose to make a little tour in the Eifel district it would be better to start from Cologne instead of Coblentz and work back down the Moselle from Trèves. Another way is to leave the Rhine at Andernach and cut across the country to Mayen and then on to Daun, where you are in the centre of this lava land. This is a good starting place, but railways will not help you being non-existent, and good walking powers are indispensable. It is a good plan to work down by Pulvermaar-Gillenfeld and Manderscheid. If you are unequal to much walking and shrink from separation between you and your luggage, you can comfortably but prosaically journey from Daun to Manderscheid by diligence in two or three hours. It only makes the journey once in the day remember. Mrs. Macquoid has written a most interesting and helpful book on this district called "In the Volcanic Eifel;" she gives much valuable information concerning roads, distances, diligences, &c. It was reading her book that decided me to make the trip.

---

### TRAVEL NOTES AND QUERIES.

PARAMÉ AND MALVERN (Wanderer).—I do not think Malvern would be very suitable for one needing an invalid chair; the expense would be about the same as at Eastbourne I should think. Paramé would be very suitable as far as the chair is concerned, because it is a flat place, but there is hardly any shade, which would be a great drawback. It is a lively place, dry and bright, but too shadeless to my mind. As to its suitability and that of Malvern for your patient you must consult a doctor; it is a most risky thing to decide such points for yourself. When he has suggested suitable resorts to you for this special case I shall be pleased to tell you anything else about hem that I can.

SOUTHERN BRITTANY (Madge).—If you really have only ten days to spare the journey is too long. The voyage to St. Malo is a night one, so that you do not lose time, but from St. Malo to Aurey cannot be done in a day unless the boat should arrive before 6 A.M. at St. Malo. Brittany trains are not quick. It seems to me too much actual travelling for such a short holiday. You can see very fine coast scenery in the extreme north of Brittany, round Perros-Guirec and Paimpol, both reached by diligence from St. Brieue.

A MONTH IN NORTH ITALY (Perseus).—Certainly it is worth while. You will reach Milan at 7.40 P.M., having left Charing Cross at 11 A.M. the previous day, so that it is not a very long journey. Stay two nights in Milan, there is more to be seen than the passer through is aware of. I think four days will do for Verona, and a week at Venice and a week at Florence will leave you time to get home comfortably. The best route for you will be Calais and Lille.

SOUTH DEVON (Johannesburg).—I know of nothing so low in price as those you mention, though, probably with diligent search such might be found. Very cheap lodgings are to be had still, I believe, at Fowey, in Cornwall, but alas! sweet simplicity is rapidly disappearing before the invading tourist. Sidmouth remains reasonable, and for a stay of a fortnight you can make good terms, but not so low as yours.

FLORENCE FOR THE WINTER (Artist).—If you are contented with humble surroundings you can certainly live comfortable in Florence for 4s. per day. The accommodation will not be luxurious, and your bedroom probably almost in the skies, but that matters nothing as you will be little in it, except for the purposes of sleeping. The Piazza Santa Spirito would be a good locality for you, not far off, but not expensive like the Lung 'Arno and the Tornabuoni, both of which are beyond humble purses. (2) No, Fiesole would not answer in any way.

